# Modeling the Molecular Impact of SARS-CoV-2 Infection on the Renin-Angiotensin System

**DOI:** 10.3390/v12121367

**Published:** 2020-11-30

**Authors:** Fabrizio Pucci, Philippe Bogaerts, Marianne Rooman

**Affiliations:** 1 Computational Biology and Bioinformatics, Université Libre de Bruxelles, CP 165/61, Roosevelt Ave. 50, 1050 Brussels, Belgium; fapucci@ulb.ac.be; 2 Biosystems Modeling and Control, Université Libre de Bruxelles, CP 165/61, Roosevelt Ave. 50, 1050 Brussels, Belgium; Philippe.Bogaerts@ulb.ac.be

**Keywords:** SARS-CoV-2, renin angiotensin system, mathematical modeling, RAS-blockers, acute respiratory distress syndrome

## Abstract

SARS-CoV-2 infection is mediated by the binding of its spike protein to the angiotensin-converting enzyme 2 (ACE2), which plays a pivotal role in the renin-angiotensin system (RAS). The study of RAS dysregulation due to SARS-CoV-2 infection is fundamentally important for a better understanding of the pathogenic mechanisms and risk factors associated with COVID-19 coronavirus disease and to design effective therapeutic strategies. In this context, we developed a mathematical model of RAS based on data regarding protein and peptide concentrations; the model was tested on clinical data from healthy normotensive and hypertensive individuals. We used our model to analyze the impact of SARS-CoV-2 infection on RAS, which we modeled through a downregulation of ACE2 as a function of viral load. We also used it to predict the effect of RAS-targeting drugs, such as RAS-blockers, human recombinant ACE2, and angiotensin 1–7 peptide, on COVID-19 patients; the model predicted an improvement of the clinical outcome for some drugs and a worsening for others. Our model and its predictions constitute a valuable framework for in silico testing of hypotheses about the COVID-19 pathogenic mechanisms and the effect of drugs aiming to restore RAS functionality.

## 1. Introduction

Since December 2019, the world has been facing a global viral pandemic of the novel severe acute respiratory syndrome coronavirus 2, “SARS-CoV-2”; this pandemic has, to date, caused millions of people to be infected and hundreds of thousands to die [1]. First detected in the city of Wuhan (China) [2,3,4,5], SARS-CoV-2 spread rapidly throughout the world. The coronavirus family, to which SARS-CoV-2 belongs, includes a number of viruses, such as SARS-CoV and MERS-CoV, which have been implicated in serious epidemics that cause acute respiratory distress syndrome (ARDS). There is not yet consensus on the origin of SARS-CoV-2 [6,7,8,9], but the primary hypothesis is that it originated from bat (*Rhinolophus affisor*) or pangolin (*Manis javanica*), since the genomes of these two viral species share high sequence identity with SARS-CoV-2.

Coronaviral genomes encode a series of structural proteins, one of which is the spike glycoprotein or S-protein that protrudes from the membrane surface [9]. Similar to the SARS-CoV virus that was identified in 2003, the S-protein of SARS-CoV-2 has been shown to bind to the angiotensin-converting enzyme 2 (ACE2), so that it can be used as an entry receptor to the cell [9,10,11,12,13]. This protein plays a pivotal role in the renin-angiotensin system (RAS) signaling pathway [14] by cleaving angiotensin I and II peptides to generate angiotensin 1–9 and the biologically active peptide angiotensin 1–7, respectively [15,16]. ACE2 is highly expressed in type II alveolar cells of lung, epithelial cells of oral mucosa, colon enterocytes, myocardial cells, and kidney proximal tubule cells. The protective role of ACE2 in severe ARDS is also widely recognized [17,18]. Indeed, it has been shown, both in in vitro and in vivo mouse models, that a loss of ACE2 expression causes increased production of angiotensin II and that this contributes to lung failure [18].

It has already been established years ago that the SARS-CoV spike protein interferes with RAS due to its binding to ACE2 [19], thus causing ACE2 downregulation; this has opened up a number of interesting means of tackling SARS-CoV infection through RAS modulation. Indeed, injection of a soluble form of recombinant human ACE2 (rhACE2, GSK2586881) into mice infected with SARS-CoV appears to have a double role [18]: it slows the viral infection by binding to the S-protein and rescues ACE2 activity, thus causing angiotensin II reduction and protecting lung from severe failure.

rhACE2 has been tested in phase II trials for its ability to ameliorate ARDS [20]. Although rhACE2 treatment is well tolerated by patients and offers a significant reduction in the angiotensin II level, clinical distress severity was not reduced in a recent pilot study [20]. Further studies are needed to understand the biological differences between the responses of animal models and humans.

Since SARS-CoV-2 also targets ACE2 receptors when it infects cells, it is logical to hypothesize that rhACE2 might help reduce the severity of COVID-19 disease [21]. Indeed, it has been shown that rhACE2 inhibits SARS-CoV-2 infection in vitro and that this inhibition depends both on the initial quantity of the virus and on the rhACE2 concentration [22]. Following these exciting results, a clinical trial with exogenous submission of rhACE2 recently started [23]. A number of other clinical trials are also underway that target the dysregulated RAS to restore its functionality [24,25,26,27,28].

**Figure 1:**
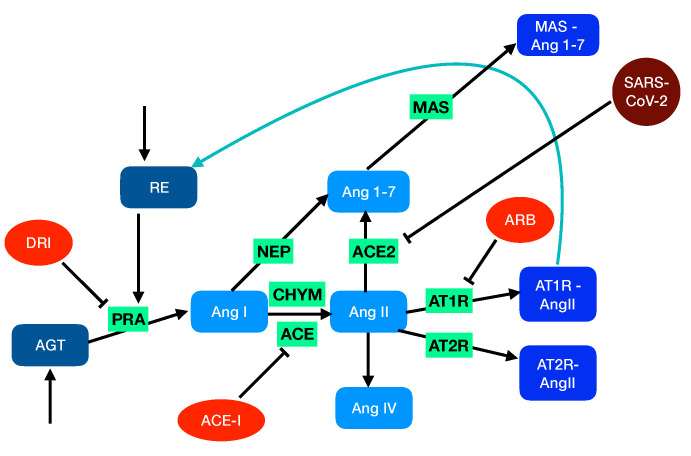
Schematic representation of RAS. In the unperturbed system, soluble proteins that are explicitly considered in the model are in blue grey, the peptides in light blue, and the peptide-bound membrane proteins in medium blue. The activities and enzymes considered only through reaction rates are in green. The feedback loop is indicated in blue. In the perturbed system, the drugs are in orange and SARS-CoV-2 in dark red.

Hypertension and cardiovascular disease have been shown to be risk factors in cases of SARS-CoV-2 infection. This brings into question what might be the potential effects on the COVID-19 development of the RAS-targeting drugs that are used to treat hypertension and cardiovascular disease. RAS-targeting drugs fall into three categories: (i) angiotensin-converting enzyme inhibitors (ACE-I), (ii) angiotensin receptor blockers (ARBs), and (iii) direct renin inhibitors (DRIs) (Figure 1). Several recent studies on large patient cohorts [29,30,31] concluded that there is only a weak correlation between treatment with drugs from these categories and any substantial increase in the risk of COVID-19.

Despite these interesting findings, there is not yet a detailed understanding of how SARS-CoV-2 infection leads to a dysregulation of RAS and, in severe cases, to ARDS. It is of fundamental importance that we gain better insights into the perturbed RAS in order to properly elucidate the pathogenic mechanisms and associated risk factors of SARS-CoV-2 infection; this, in turn, will enable novel therapeutic strategies to be designed and tested so that disease progression can be inhibited.

## 2. Methods

### 2.1. Modeling the Renin-Angiotensin System

RAS has been widely studied both experimentally [32,33,34] and computationally [35,36,37,38]. It plays a key role in the regulation of a large series of physiological systems including the renal, lung, and cardiovascular systems. Consequently, its dysregulation is related to multiple pathological conditions such as hypertension and ARDS, just to mention a few [39,40,41,42,43].

There are two different types of RAS: the circulating RAS that is localized in the plasma and is involved in the regulation of the cardiovascular system and the tissue-localized systems that act intracellularly or interstitially within different organs in association with the systemic RAS or independently of it. Here, we focus on the local RAS within the pulmonary circulation and model its network of biochemical reactions, as schematically depicted in Figure 1.

When the blood pressure decreases, the juxtaglomerular kidney cells that sense changes in renal perfusion pressure secret an aspartic protease protein called renin (RE, EC 3.4.23.15). The activity of this enzyme, called plasma renin activity (PRA), is the common measure used in clinical practice to set up the diagnosis and treatment design of essential hypertension.

The dynamics of the renin concentration can be modeled as:

(1) $$\begin{array}{c} \frac{d[\mathrm{RE}]}{dt}=\beta -\frac{\mathrm{Log}\;2}{h_{re}}\left[\mathrm{RE}\right] \end{array}$$

where $h_{re}$ is renin’s half-life and β its production rate. The latter is not constant, but depends on other elements of RAS, which we will discuss later in the section. The role of renin is to cleave the N-terminus of a protein from the serine protease inhibitor family called angiotensinogen (AGT) to form the decapeptide hormone angiotensin I (AngI). The dynamics of the angiotensinogen can be written as:

(2) $$\frac{d[\mathrm{AGT}]}{dt}=k_{agt}-c_{re}\left[\mathrm{RE}\right]-\frac{\mathrm{Log}\;2}{h_{agt}}\left[\mathrm{AGT}\right]$$

where the reaction rate $c_{re}$ relates the renin concentration to its activity, $k_{agt}$ is AGT’s production rate, and $h_{agt}$ its half-life.

The AngI peptide is further cleaved by different enzymes:

- The angiotensin-converting enzyme (ACE, EC3.4.15.1) is a zinc metalloproteinase located mainly in the capillaries of lung and in the endothelial cells. It catalyzes the transformation of AngI into the octapeptide angiotensin II (AngII).
- Chymase (CHY, EC 3.4.21.39), a serine protease that is mainly localized in blood vessels and heart, also catalyzes the transformation of AngI into AngII.
- Neprilysin (NEP, EC3.4.24.11), another zinc metalloproteinase that is expressed in a wide variety of tissues, catalyzes the transformation of AngI into the heptapeptide hormone angiotensin-(1-7) (Ang1-7).

The dynamics of AngI can thus be described as:

(3) $$\begin{array}{c} \frac{d[\mathrm{AngI}]}{dt}=c_{re}\left[\mathrm{RE}\right]-\left(c_{ace}+c_{chy}+c_{nep}\right)\left[\mathrm{AngI}\right]-\frac{\mathrm{Log}\;2}{h_{angI}}\left[\mathrm{AngI}\right] \end{array}$$

where $c_{ace}$, $c_{chy}$, and $c_{nep}$ are the reaction rates associated with the corresponding enzymatic reactions.

The role of AngII in RAS is central since it has a vasoconstriction effect, enhances blood pressure, and triggers inflammatory processes and fibrosis. In lung, the capillary blood vessels are among the sites that have the highest ACE expression and production of AngII. Its dysregulation has frequently been related to a wide series of chronic and acute diseases such as pulmonary fibrosis and ARDS.

AngII’s effects are mediated by two G-protein coupled receptors (GPCR) called angiotensin II type 1 (AT1R) and type 2 (AT2R). In addition, it can be cleaved by different enzymes. For example, ACE2 generates Ang1-7 peptides, and aminopeptidase A (APA, EC 3.4. 11.7) generates other peptides such as angiotensin III (AngIII), which is further cleaved to AngIV. In our model, we skipped all the details about the enzymatic reactions AngII-AngIII-AngIV and kept only a single equation for their transformation. The dynamics of AngII and AngIV can thus be written as:

(4) $$\begin{array}{ccc} \frac{d[\mathrm{AngII}]}{dt} & = & (c_{ace}+c_{chy})\left[\mathrm{AngI}\right]\\ & & -\left(c_{ace2}+c_{angIV}+c_{at1r}+c_{at2r}\right)\left[\mathrm{AngII}\right]-\frac{\mathrm{Log}\;2}{h_{angII}}\left[\mathrm{AngII}\right] \end{array}$$

(5) $$\frac{d[\mathrm{AngIV}]}{dt}=c_{angIV}\left[\mathrm{AngII}\right]-\frac{\mathrm{Log}\;2}{h_{angIV}}\left[\mathrm{AngIV}\right]$$

where $h_{angII}$ and $h_{angIV}$ are the half-lives of the peptides and $c_{ace2}$, $c_{angIV}$, $c_{at1r}$, and $c_{at2r}$ the rates of the enzymatic reactions.

The dynamics of the peptide-bound form of the GPCRs are expressed as:

(6) $$\frac{d[\mathrm{AT}1R-\mathrm{AngII}]}{dt}=c_{at1r}\left[\mathrm{AngII}\right]-\frac{\mathrm{Log}\;2}{h_{at1r}}\left[\mathrm{AT}1R-\mathrm{AngII}\right]$$

(7) $$\frac{d[\mathrm{AT}2R-\mathrm{AngII}]}{dt}=c_{at2r}\left[\mathrm{AngII}\right]-\frac{\mathrm{Log}\;2}{h_{at2r}}\left[\mathrm{AT}2R-\mathrm{AngII}\right]$$

where [AT1R-AngII] and [AT1R-AngII] are the concentrations of the bound forms of the receptors and $h_{at1r}$ and $h_{at2r}$ their half-lives.

Until now, we have modeled the ACE/AngII/AT1R regulatory axis of RAS. Since the last two decades, it became clear that there is another RAS axis that acts as a counterregulator of the first axis [44]. The key role of this second axis is played by the Ang1-7 peptide that is mainly produced from AngII by the ACE2 enzyme and binds to the transmembrane GPCR called MAS. However, Ang1-7 can also be obtained as an enzymatic product from AngI via the catalytic activity of NEP and, to a lesser extent, from Ang1-9 via ACE and NEP. We overlooked the Ang1-9-related enzymatic reactions in our model, as they contribute less to Ang1-7 formation [33,34]. The dynamical equations for the Ang1-7 peptide and the MAS-bound receptor are as follows:

(8) $$\begin{array}{c} \frac{d[\mathrm{Ang}1-7]}{dt}=c_{nep}\left[\mathrm{AngI}\right]+c_{ace2}\left[\mathrm{AngII}\right]-c_{mas}\left[\mathrm{Ang}1-7\right]-\frac{\mathrm{Log}\;2}{h_{ang1-7}}\left[\mathrm{Ang}1-7\right] \end{array}$$

(9) $$\frac{d[\mathrm{MAS}-\mathrm{Ang}1-7]}{dt}=c_{mas}\left[\mathrm{Ang}1-7\right]-\frac{\mathrm{Log}\;2}{h_{mas}}\left[\mathrm{MAS}-\mathrm{Ang}1-7\right]$$

Let us now go back to Equation (1) in which we simply expressed the renin production as a baseline term β. To describe the autoregulatory nature of RAS, this term has to depend on the production of other species, thus introducing a feedback regulation. It is known that this feedback depends on AT1R bound to AngII. Following other models [37,38], we express β as::

(10) $$\beta =\beta_{0}+\left({\left(\frac{{\left[\mathrm{AT}1R-\mathrm{AngII}\right]}_{0}^{N}}{\left[\mathrm{AT}1R-\mathrm{AngII}\right]}\right)}^{\delta }-1\right)$$

where $\beta_{0}$ is a constant parameter to be identified and ${\left[\mathrm{AT}1R-\mathrm{AngII}\right]}_{0}^{N}$ the equilibrium concentration for healthy normotensive humans. δ is a positive number that we fixed to 0.8 [37].

Technical details on the procedure used to solve the model and on model stability are given in Section 2.6 and Section 2.7.

### 2.2. Modeling Blood Pressure

Blood pressure is well known to be increased by the concentration of AngII bound to AT1R. It has also been described to be decreased by the concentration of MAS bound to Ang1-7 and of AT2R bound to AngII, but the precise mechanism is not yet known [45,46,47]. Therefore, we did not introduce in our model a feedback between these concentrations and renin production, as we did for AT1R-AngII, and modeled the diastolic blood pressure (DBP) simply from the AT1R-AngII concentration:

(11) $$DBP=P_{0}+P_{1}\left[\mathrm{AT}1R-\mathrm{AngII}\right]$$

We identified the two parameters $P_{0}$ and $P_{1}$ by fixing DBP equal to 80 mmHg for normotensive individuals and to 110 mmHg for hypertensive individuals. Hence, $P_{0}+P_{1}{\left[\mathrm{AT}1R-\mathrm{AngII}\right]}_{0}^{N}=80$ mmHg and $P_{0}+P_{1}{\left[\mathrm{AT}1R-\mathrm{AngII}\right]}_{0}^{H}=110$ mmHg, where the *N* and *H* superscripts denote the concentration in normotensive and hypertensive individuals and the 0 subscript the equilibrium concentrations.

### 2.3. Modeling RAS-Blocker Effects

Since dysregulated RAS with high levels of AngII is related to essential hypertension, a wide range of RAS-targeting drugs have been developed in the last forty years [48]. They can be classified into three different categories based on their pharmacological target [49]:

- Angiotensin-converting enzyme inhibitors (ACE-I) that bind to ACE and thus inhibit the formation of angiotensin II and the associated vasoconstriction and inflammatory cascades. Examples of this type of drug are enalapril, lisinopril, and captopril.
- Angiotensin receptor blockers (ARB) that block the binding of AngII to AT1R and thus act in antagonism with AngII. Examples are candesartan, losartan, and valsartan.
- Direct renin inhibitors (DRI) that act on renin and thus inhibit the conversion of AGT to AngI. Examples are aliskiren, enalkiren, and remikiren.

We modeled the action of these three types of drugs by modifying the reaction rates associated with their targets as:

(12) $$\begin{array}{c} c_{ace}⟶c_{ace}\times \left(1-\gamma_{\mathrm{ACE}-I}\right)\\ c_{at1r}⟶c_{at1r}\times \left(1-\gamma_{\mathrm{ARB}}\right)\\ c_{re}⟶c_{re}\times \left(1-\gamma_{\mathrm{DRI}}\right) \end{array}$$

where $\gamma_{\mathrm{ACE}-I}$, $\gamma_{\mathrm{ARB}}$, and $\gamma_{\mathrm{DRI}}$ are parameters describing the drug activity.

### 2.4. Modeling SARS-CoV-2 Infection

Since ACE2 is the entry point of SARS-CoV-2 [19], it is downregulated upon infection, and this impacts substantially the local and systemic RASs. In order to model the downregulation effect due to the virus, we modified the ACE2 reaction rate with the function $\gamma_{\mathrm{CoV}}$:

(13) $$c_{ace2}⟶c_{ace2}\times \left(1-\gamma_{\mathrm{CoV}}\left(C_{t}\right)\right)$$

We chose $\gamma_{\mathrm{CoV}}$ to be a sigmoid function of the cycle threshold value $C_{t}$, which is inversely related to the viral load [50]:

(14) $$\gamma_{\mathrm{CoV}}=\frac{1}{1+e^{aC_{t}-b}}$$

where *a* and *b* are positive real numbers. $C_{t}$ values of 31.5, 27.6, and 23.8 correspond to mild, moderate, and severe disease, respectively, and $C_{t}>40$ to undetected viral infection [51]. We thus chose the inflection point of the sigmoid at $C_{t}=31.5$ and imposed $\gamma_{\mathrm{CoV}}>0.99$ for $C_{t}>40$. Using these relations, we identified the values of the parameters *a* and *b*. They are reported in Table 1.

### 2.5. Modeling ARDS Severity

To model ARDS severity and how the lungs of SARS-CoV-2 patients evolve in response to RAS dysregulation, we introduced a phenomenological relation to estimate the PaO2/FiO2 ratio, defined as the ratio between the partial pressure of arterial oxygen (PaO2) and the fraction of inspired oxygen (FiO2). This quantity plays a key role in the assessment of ARDS patients [52,53]. The normal range of PaO2/FiO2 is between 400 and 500 mmHg. Mild and moderate ARDS are characterized by PaO2/FiO2 values in the range [200–300] mmHg and [100–200] mmHg, respectively. ARDS is severe for values below 100 mmHg.

We predicted the PaO2/FiO2 ratio as a function of the AngII and Ang1-7 concentrations:

(15) $$PAO2/FIO2=A_{0}+A_{1}\left(-\frac{\left[\mathrm{AngII}\right]}{{\left[\mathrm{AngII}\right]}_{0}}+\frac{\left[\mathrm{Ang}1-7\right]}{{\left[\mathrm{Ang}1-7\right]}_{0}}\right)$$

where $A_{0}$ and $A_{1}$ are two parameters that we identified on the basis of our model by comparing the baseline RAS with the same system in which ACE2 is knocked out. In the former case, we fixed PaO2/FiO2 = 450 mmHg and, in the latter, PaO2/FiO2 = 50 mmHg.

### 2.6. Solving the RAS Model

The mathematical model of RAS described in Equations (1)–(10) is a system of ordinary differential equations (ODEs), which are linear except for the feedback loop of Equation (10).

We collected from the literature the values of the equilibrium concentrations of all proteins and peptides except renin and MAS bound to Ang1-7, for normotensive and hypertensive humans (Table 2). From these values, we fixed the parameters that appear in the phenomenological relations (11) and (15) for DBP and PaO2/FiO2 (Table 1). We also collected the values of the half-life of all proteins and peptides but MAS; we assumed the latter to be equal to that of the other membrane receptors (Table 1). Moreover, we estimated the value of reaction rate $c_{re}$ from [36,54].

Using these concentration and parameter values, we solved the system of nine ODEs (Equations (1)–(9)) in the stationary state to identify the unknown parameters and concentrations. However, these equations have 12 unknowns: $k_{agt}$, $\beta_{0}$, $c_{ace}$, $c_{ace2}$, $c_{angIV}$, $c_{at1r}$, $c_{at2r}$, $c_{mas}$, $c_{chy}$, $c_{nep}$, [RE]${}_{0}$, and [MAS-Ang1-7]${}_{0}$. We had thus to assume three additional relations, which are:

(16) $$\begin{array}{ccc} c_{mas} & = & c_{at2r} \end{array}$$

(17) $$\begin{array}{ccc} c_{chy} & = & 0 \end{array}$$

(18) $$\begin{array}{ccc} c_{nep} & = & 0 \end{array}$$

Since no quantitative data related to the MAS receptor can be found in the literature, we hypothesized the first relation assuming MAS and AT2R to be equally expressed and the affinity of Ang1-7 for MAS to be similar to the affinity of AngII for AT2R [46]. Moreover, we assumed $c_{chy}=0$ and $c_{nep}=0$, but discuss the effect of non-vanishing values in Section 4.

By imposing these three additional relations, we solved the system of nine ODEs in the stationary state. The values obtained for [RE]${}_{0}$, [MAS-Ang1-7]${}_{0}$, $k_{agt}$, $\beta_{0}$, $c_{ace}$, $c_{ace2}$, $c_{angIV}$, $c_{at1r}$, and $c_{at2r}$ for normotensive and hypertensive humans, are given in Table 2.

### 2.7. Stability of the RAS Model

The system of nine ODEs (Equations (1)–(9)) can be summarized in the form:

(19) $$\frac{dx(t)}{dt}=f\left(x\left(t\right),\theta \right)$$

where x(t) is the vector containing the nine state variables, i.e., the concentrations of all proteins and peptides at time *t*, θ is the vector with all the production, kinetic, and half-life parameters, and *f* represents the vector that corresponds to the right-hand sides of Equations (1)–(9). In order to analyze the stability of the two steady states $x_{0}^{N}$ and $x_{0}^{H}$ for normotensive and hypertensive individuals, respectively, we computed the eigenvalues of the Jacobian matrix:

(20) $$J\left(x_{0}\right)=\frac{\partial f(x,\theta )}{\partial x}{|}_{x=x_{0}}$$

where $x_{0}$ stands for either $x_{0}^{N}$ or $x_{0}^{H}$.

In both the normotensive and hypertensive cases, seven strictly negative real values were obtained, together with two complex conjugate eigenvalues with strictly negative real parts. Both steady states $x_{0}^{N}$ and $x_{0}^{H}$ are therefore stable. The nonzero imaginary parts of the two complex conjugate eigenvalues are responsible for some damped oscillations in transient responses to parameter changes, but the overshoots are limited. It is interesting to note that the imaginary part is more than three times lower in the hypertensive case, hence leading to more damped responses in comparison with the normotensive case.

To quantify the state variable transients and the aforementioned overshoots, we simulated step responses corresponding to a 10% increase in the normal baseline for renin production $\beta_{0}$. We observed some damped oscillations during the transient phase of the normotensive case, with very limited overshoots, e.g., 1.3% for the RE concentration. In the hypertensive case, the imaginary part of the complex conjugate eigenvalues is so low that the overshoots become almost undetectable (0.025%).

## 3. Results

The main objective of this paper is to investigate the effect of RAS-targeting drugs and SARS-CoV-2 infection, both individually and in combination, on the RAS of normotensive and hypertensive individuals. The robustness and predictive power of our model was first assessed by investigating the effects on RAS of three types of antihypertensive drugs: (i) ACE-I, (ii) ARB, and (iii) DRI (described in Section 2.3). This assessment included a comparison of model simulations with patient clinical data. Following the confirmation of model robustness and accuracy, ACE2 downregulation due to viral infection was introduced into the model to quantitatively predict how RAS is perturbed in COVID-19.

### 3.1. Model Predictions and Clinical Data on RAS-Blocker Drugs

**Figure 2:**
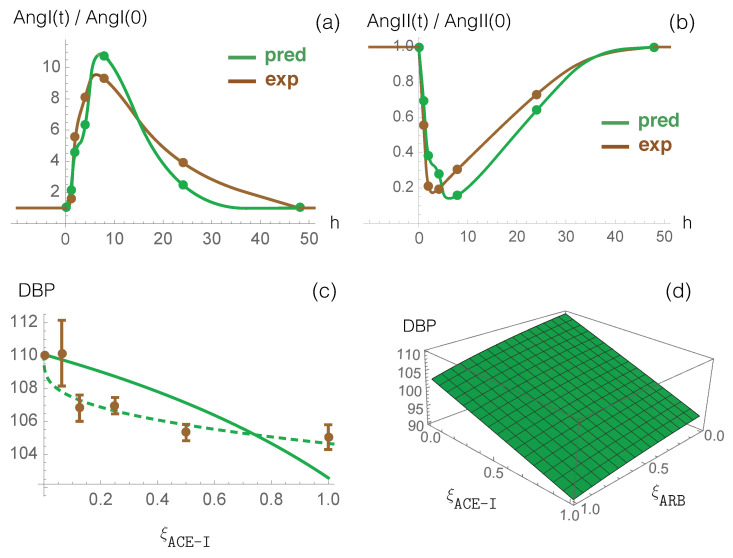
Dynamical response of RAS to ACE-I (enalapril) administration. Comparison between the computational prediction (green) and the experimental data (brown) of normalized AngI (**a**) and AngII (**b**) as a function of time (in hours) after the single dose administration. Continuous lines are obtained through data interpolation. (**c**) Measured DBP averaged over more than ten ACE-I types as a function of the normalized dosage $\xi_{\mathrm{ACE}}-I$ (dosage divided by maximal dosage) (brown points) and predicted DBP as a function of $\xi_{\mathrm{ACE}}-I$ values considering $\gamma_{\mathrm{ACE}-I}=0.5\;\xi_{\mathrm{ACE}-I}$ (continuous green line) and $\gamma_{\mathrm{ACE}-I}=0.4\;\xi_{\mathrm{ACE}-I}^{1/4}$ (dashed green line). (**d**) Predicted effect of the combination of ACE-I and ARB on DPB values as a function of the normalized drug dosages $\xi_{\mathrm{ACE}-I}$ and $\xi_{\mathrm{ARB}}$, considering $\gamma_{\mathrm{ACE}-I}=0.5\;\xi_{\mathrm{ACE}-I}$ and $\gamma_{\mathrm{ARB}}=0.5\;\xi_{\mathrm{ARB}}$.

The effect of enalapril, an ACE-I type drug, on plasma ACE activity and on plasma levels of AngI and AngII has been measured in normotensive individuals who received a single oral dose of 20 mg [60]. To compare these data with model predictions, we first fitted the $\gamma_{\mathrm{ACE}-I}$ parameter introduced in Equation (12) to the ACE activity values during enalapril administration divided by the pre-treatment activity (measured by an antibody-trapping assay). Once $\gamma_{\mathrm{ACE}-I}$ was set, we used our model to predict the dynamical response of RAS to this inhibitor drug. The time-dependent values of the AngI and AngII concentrations, normalized by their concentration at time 0, are shown in Figure 2a,b, both for our model predictions and experimental enalapril data; there is very good agreement between the two curves, without any further parameter fitting. The excellent correspondence between model prediction and experimental data is also clear from the root mean squared deviation (rmsd) between model prediction and experimental data on all time points following drug administration, as shown in Table 3.

Our model, thus, captures the known dynamics of ACE inhibition, (i.e., increased AngI levels and decreased AngII levels); this has the effect of lowering the concentration of AngII bound to AT1R and, thus, also lowers the blood pressure (Equation (11)).

To study the effect of ARB antihypertensive drugs on RAS, we considered data from [61], which measured the effects of different types of AT1R blocking molecules on the plasma levels of AngII in normotensive individuals. Specifically, the study participants received a single 50 mg dose of losartan, 80 mg of valsartan, or 150 mg of irbesartan. First, we fitted the $\gamma_{\mathrm{ARB}}$ parameter (defined in Equation (12)) to the in vitro ability of the administered drug to induce the AngII receptor blockade, as measured by an AT1R radioreceptor binding assay [61]. We then used our model to predict the time-dependent AngI level, which was normalized by its concentration prior to drug administration. The results were evaluated through the rmsd between experimental and predicted values of AngI/AngI${}_{0}$ at different time points after drug administration. The results, which are detailed in Table 3, clearly show that our model accurately predicts the RAS response to ARBs.

We also studied the effect of DRI-type drugs using experimental data that describe PRA activity and RE, AngI, and AngII concentrations, when different doses of aliskiren were administered orally to normotensive individuals [62]. We used the PRA activity data to fit the $\gamma_{\mathrm{DRI}}$ parameter (introduced in Equation (12)), and we used our model to calculate the normalized AngI and AngII levels as a function of time. Here also, the results from our model and the experimental concentration data agree very well, as shown in Table 3.

In summary, the rmsd between predicted and experimental values of normalized AngI and AngII levels, averaged over all tested drugs, dosages, and a total of 38 time points, is 0.57 and 0.18, respectively (Table 3). These values should be compared with average experimental values of 1.7 and 0.5, respectively, demonstrating excellent agreement between experimental data and model predictions.

It should be noted that all reported experimental data were obtained after administration of single doses of RAS-targeting drugs. However, for hypertensive patients receiving long-term treatment, the expression of some enzymes involved in RAS could be either up- or down-regulated; we will return to this point in Section 4.

Finally, we compared model predictions against clinical data from large cohorts of patients describing the effect of ACE-I and ARB drug administration on blood pressure [63,64]. We first analyzed the response to ACE-I drugs alone. We plot measured DBP values averaged over more than ten ACE-I drug types as a function of the normalized dosage $\xi_{\mathrm{ACE}-I}$ [63] in Figure 2c, as well as predicted DBP values. We first considered a linear relation between $\gamma_{\mathrm{ACE}-I}$ and the normalized drug dosage ($\gamma_{\mathrm{ACE}-I}=0.5\;\xi_{\mathrm{ACE}-I}$). Despite this simplification, chosen to limit the number of parameters and thus overfitting issues, the curve reproduces the experimental data reasonably well. We also defined a non-linear relationship between these two quantities by introducing additional parameters: $\gamma_{\mathrm{ACE}-I}=0.4\;\xi_{\mathrm{ACE}-I}^{1/4}$. We thus obtained a better fit as shown in Figure 2c.

We then studied the effect of the combined administration of the two drugs, ARB and ACE-I, on blood pressure, plotting the predicted DBP values as a function of both $\xi_{\mathrm{ACE}-I}$ and $\xi_{\mathrm{ARB}}$ (see Figure 2d). We found that combined administration of ARB and ACE-I reduces DBP by 4 mmHg when compared with ARB monotherapy and by 12 mmHg when compared with ACE-I monotherapy. These predictions should be compared with clinical DBP values of 3 mmHg for combined administration compared to either monotherapy [64]. Thus, our model again provides an excellent prediction of experimental clinical data; further improvements to the model’s predictive strength are possible by fixing the $\gamma_{\mathrm{ARB}}$ value at the maximum dose to be slightly lower than the corresponding $\gamma_{\mathrm{ACE}-I}$ value.

### 3.2. RAS in COVID-19

It is known that ACE2 is the cellular receptor of the spike glycoprotein of SARS-CoV-2 [9,10,11,12,13] and that it triggers the entry of SARS-COV-2 into the host cell. Although ACE2 is expressed in a variety of tissues [65,66,67], it is expressed mainly in the alveolar epithelial cells of lung, in the gastrointestinal tract, and in the kidney proximal tubular cells.

**Figure 3:**
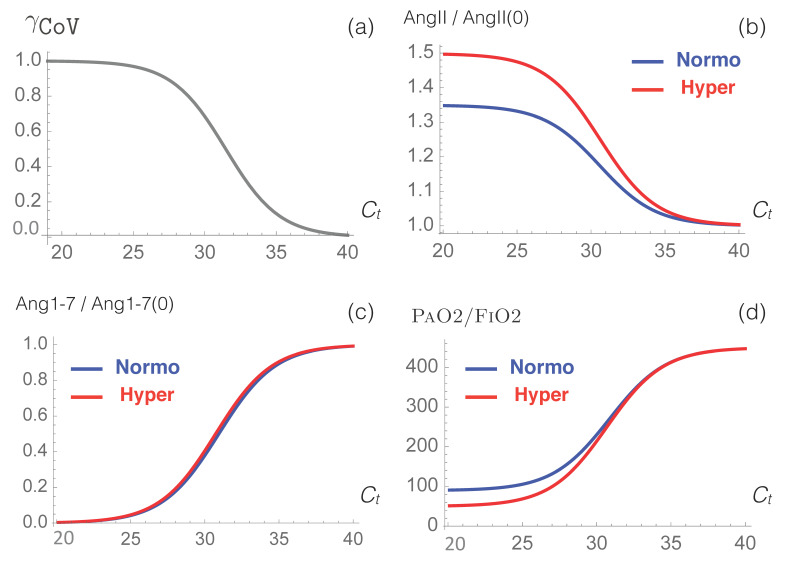
Simulated response of RAS to viral infection. (**a**) The $\gamma_{\mathrm{CoV}}$ function used to model the effect of the infection as a function of $C_{t}$, the cycle threshold of the virus. (**b**–**d**) Predictions obtained from our model for the normalized levels of AngII and Ang1-7 and for the physiological PaO2/FiO2 value, as a function of $C_{t}$, for normotensive (blue) and hypertensive (red) individuals.

Here, we used our model to predict how RAS is perturbed by the SARS-CoV-2 virus. Simulation results of AngII and Ang1-7 concentrations and of the physiological value of PaO2/FiO2 as a function of SARS-CoV-2 viral load are presented in Figure 3 and in Table 4.

We observe that the AngII level increases with increasing viral load, with a much stronger increase for hypertensive than for normotensive patients. The AngII level is predicted to increase by approximately 15% for patients with moderate and severe COVID-19 (Table 4); this prediction is in very good agreement with the experimental value of 16% found in [68], but in poorer agreement with the value of 35% resulting from a study of only 12 patients [69].

We also observe that our model predicts a severe reduction of the Ang1-7 level, due to ACE2 downregulation; this reduction is the same for hypertensive and normotensive patients.

The overall result of the model is that RAS becomes imbalanced upon SARS-CoV-2 infection, with the harmful AngII axis upregulated and the counteracting Ang1-7 axis severely downregulated. This imbalance can be related to multiple clinical manifestations of COVID-19. More specifically, increased AngII levels cause hyperinflammation, which, in turn, increases plasma proinflammatory cytokine levels (in particular, IL-6) [70,71]. In addition, thrombotic events are observed, since AngII promotes the expression of plasminogen activator inhibitor-1 (PAI-1) and tissue-factors (TFs) [72,73]. Ang1-7, which normally counteracts these various effects [44], is downregulated by SARS-CoV-2 infection, such that COVID-19 clinical manifestations become increasingly severe as the disease develops.

Moreover, our model predicts severe ARDS with PaO2/FiO2 < 100 mmHg for normotensive and hypertensive patients whose $C_{t}$ values are smaller than 24.1 and 27.0, respectively. Our model predicts moderate ARDS, characterized by a PaO2/FiO2 ratio in the range of 100-200 mmHg, for normotensive and hypertensive patients having $24.1<C_{t}<29.3$ and $27.0<C_{t}<29.7$, respectively, and mild ARDS, characterized by a PaO2/FiO2 ratio in the range of 200–300 mmHg for normotensive and hypertensive patients having $29.3<C_{t}<31.4$ and $29.7<C_{t}<31.6$, respectively.

Our modeling approach suggests a weak relationship between hypertension and ARDS severity resulting from SARS-CoV-2 infection. The mean value of the PAO2/FIO2 ratio over the entire $C_{t}$ range is approximately 20 mmHg lower for hypertensive than for normotensive patients. Indeed, the large difference in AngII levels between normotensive and hypertensive patients is partially compensated by the absence of any difference in Ang1-7 levels.

### 3.3. Impact of RAS-Modulating Drugs on COVID-19 Severity

We analyzed the effect of administering a selection of drugs to normotensive and hypertensive patients who were infected with SARS-CoV-2. More specifically, we considered RAS-blocking drugs that are already commonly used to treat hypertension, as well as drugs that are currently undergoing clinical trials in the context of COVID-19, such as rhACE2 and Ang1-7.

**Figure 4:**
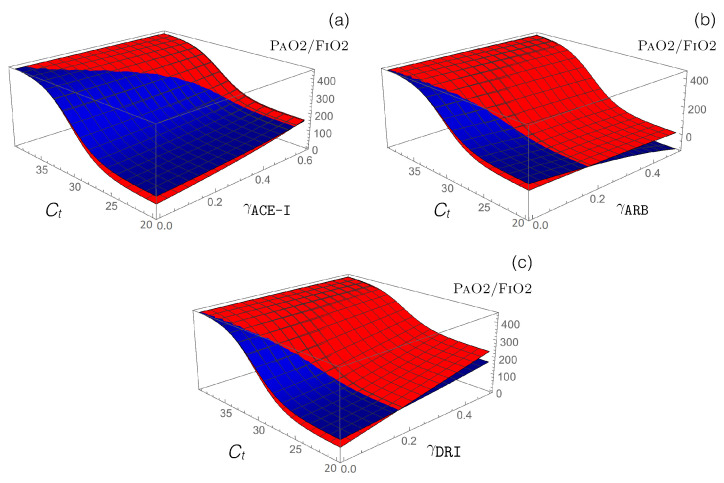
Impact of different RAS-blocking drugs in normotensive (blue) and hypertensive (red) SARS-CoV-2 patients. Predicted PAO2/FIO2 value as a function of the cycle threshold value $C_{t}$ and (**a**) $\gamma_{ACE-I}$, (**b**) $\gamma_{ARB}$, and (**c**) $\gamma_{DRI}$ functions that model the administration of the corresponding drugs.

- Antihypertensive RAS-blocking drugs: We combined the effect of each of the three RAS-blocking ACE-I, ARB, and DRI drugs, which were modeled by the enzyme-inhibiting γ functions (introduced in Equation (12)), with the ACE2-inhibiting $C_{t}$-dependent $\gamma_{\mathrm{CoV}}$ function (defined in Equation (14)), which mimics SARS-CoV-2 infection. the PaO2/FiO2 values predicted by our model are presented in Figure 4.

Our model predicts that administration of ACE-I and DRI drugs protect from the adverse effects of ARDS, especially for hypertensive patients, while ARB drugs are predicted to worsen ARDS severity, especially for normotensive patients.

Model predictions for ACE inhibitors are in agreement with clinical data, which indicate that treatment with ACE inhibitors is associated with better survival among COVID-19 patients [31,74]. Indeed, only 3% of non-surviving COVID-19 patients that were monitored were treated with ACE-I drugs compared to 9% of surviving COVID-19 patients [31]. Moreover, in a meta-analysis [74], hypertensive patients treated with ACE-I drugs were associated with a reduced mortality of 35% when compared to patients who were not treated with ACE-I drugs. in another clinical analysis [75], older patients who were treated with ACE-I drugs had a 40% lower risk of hospitalization than those who were not treated with ACE-I drugs.

No data are currently available to validate our model prediction that COVID-19 attenuation due to ACE-I drug treatment is stronger in hypertensive than in normotensive patients. Furthermore, no data are currently available to validate our model prediction that DRI and ACE-I drug treatments cause similar levels of COVID-19 disease attenuation.

In contrast to DRI and ACE-I drugs, our model predicts that ARB drug treatment worsens COVID-19 severity, with the effect being stronger for normotensive compared to hypertensive patients. Here, the agreement between model predictions and clinical data is less clear, with some clinical data in agreement with our model prediction [31,75], while other clinical data suggest that ARB drug treatment does not affect hospitalization risk [75] or mortality [74,76]. This lack of agreement must be further investigated with additional clinical data.

Moreover, we performed a quantitative prediction of the drug effects on disease severity by calculating the RAS peptide concentrations, PaO2/FiO2 values, and DPB for moderate COVID-19 patients. The results are presented in Table 5.

Administration of ACE-I drugs, modeled by $\gamma_{ACE-I}=0.5$, increases the PAO2/FIO2 value by approximately 50 and 70 mmHg for normotensive and hypertensive patients, respectively. an equivalent administration of DRI drugs increases this ratio even more, by 70 and 150 mmHg, while ARB administration decreases it by 140 and 30 mmHg for normotensive and hypertensive patients, respectively.

The opposite effect of ARBs administration compared to ACE-I and DRI drugs can be attributed to the substantial increase in AngII concentration, which is only partially balanced by a relatively small increase in Ang1-7 concentration, given that ACE2 is downregulated in SARS-CoV-2 infection.

Note that a number of ARB drugs, including valsartan and losartan, are currently being tested in clinical trials, with the hope that they will rescue RAS in COVID-19 patients [25,26,27]. Our model predicts that this will not be the case.

Finally, as shown in Table 5, the blood pressure is predicted to be unaffected by the administration of either ACE-I, ARB, or DRI to normotensive COVID-19 patients, but to be reduced by approximately 10–20 mmHg by administration to hypertensive patients.

- Other RAS-targeting drugs: We used our model to test the potential of other drugs that are currently in clinical trials to restore the functional activity of the perturbed RAS upon viral infection. First, we modeled how the administration of an exogenous supplement of rhACE2 (GSK2586881) affects RAS by modifying the reaction rate $c_{ace2}$ defined in Equation (13). This rate already includes the function $\gamma_{\mathrm{CoV}}$ that mimics SARS-CoV-2 infection, and we simply added a second function $\gamma_{\mathrm{rhACE}2}$ associated with the effects of rhACE2 administration:
  (21) $$c_{ace2}⟶c_{ace2}\times \left(1+\gamma_{\mathrm{rhACE}2}-\gamma_{\mathrm{CoV}}\left(C_{t}\right)\right)$$

**Figure 5:**
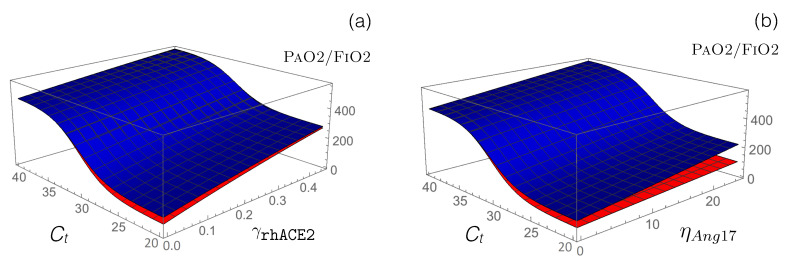
Impact on the PAO2/FIO2 value of the administration of rhACE2 and Ang1-7 in normotensive (blue) and hypertensive (red) SARS-CoV-2 patients. (**a**) Predicted PAO2/FIO2 values as a function of $C_{t}$ and $\gamma_{\mathrm{rhACE}2}$. (**b**) Predicted PAO2/FIO2 values as a function of $C_{t}$ and $\eta_{Ang17}$.

Our model predicts an increase in PAO2/FIO2 following the administration of exogenous rhACE2, thus predicting an alleviation of disease severity, as shown in Figure 5 and Table 5. Specifically, PAO2/FIO2 is predicted to increase by approximately 200 mmHg when $\gamma_{\mathrm{rhACE}2}$ is fixed at 0.5. Our model also predicts, as expected, a reduction in AngII level and an increase in Ang1-7 level.

These predictions are in agreement with both animal and in vitro studies [18,22], whereby rhACE2 is observed to alleviate virus-related ARDS severity through a double action. First, by rhACE2 binding to the virus spike protein, interaction with endogenous ACE2 is prevented, and infection is slowed down. Second, rhACE2 administration increases ACE2 activity, thus causing a reduction in AngII level and an increase in Ang1-7 level; this protects lung against severe failure.

Current clinical trial data concerning the administration of different doses of rhACE2 (0.1, 0.2, 0.4, and 0.8 mg/kg) to SARS-CoV patients at different time intervals (2, 4, and 18 h) are only in partial agreement with our model predictions [20]. Specifically, while clinical data followed the predicted decrease in [AngII] and the predicted increase in [Ang1-7], there was no sustained increase in PAO2/FIO2 compared with placebo. It has been suggested that the drug concentrations used in these clinical trials were too low to have a measurable effect on the respiratory system and that drug administration via infusion would have been more sustained [20]. More experimental and clinical data are clearly needed to further investigate the effect of rhACE2 on coronavirus-related ARDS.

Another method of boosting the second RAS axis, ACE2/Ang1-7/MAS, which is downregulated by SARS-CoV-2 infection, is to administer Ang1-7 peptides as a means of triggering anti-inflammatory and anti-fibrotic mechanisms. We modeled Ang1-7 peptide administration by introducing a new parameter, the production rate $\eta_{Ang17}$, to the dynamical Equation (8) of [Ang1-7]; this allows the model to describe the exogenous Ang1-7 level, which is added to the endogenous Ang1-7 baseline. as shown in Figure 5b and Table 5, our model predicts a clear alleviation of COVID-19 severity, with PAO2/FIO2 increasing by 50 and 130 mmHg for hypertensive and normotensive patients, respectively, upon administration of $\eta_{Ang17}=$ 25 fmol/(mL min) Ang1-7 in infusion. Note that COVID-19 alleviation is significantly stronger in normotensive compared to hypertensive patients for the same drug concentrations; a slightly stronger concentration of Ang1-7 must be administered to hypertensive patients for an equivalent effect.

Our model predicts a quantitative reduction in ARDS severity in COVID-19 patients, in agreement with the known anti-inflammation and anti-fibrosis nature of Ang1-7. Model predictions nicely agree with data from animal studies without the need for any additional fitting. For example, administration of Ang1-7 by infusion to acid-injured rats suffering from ARDS increases the baseline Ang1-7 level by a factor 2.5, leading to an increase in PAO2/FIO2 of approximately 70 mmHg [77]. However, while the PAO2/FIO2 value increases linearly in our model as a function of Ang1-7 concentration, it reaches a plateau in rats; this suggests that our model is probably oversimplified, since PAO2/FiO2 is not a linear function of Ang1-7 concentration. Further work on this aspect of our model will be possible when more data become available.

## 4. Discussion

The spike protein of SARS-CoV-2 interferes with RAS by binding to the ACE2 receptor, a key element of RAS. Despite recent progress in understanding COVID-perturbed RAS and how its functionality can be restored, more work is urgently needed in the context of the current COVID-19 pandemic.

We here present a simple computational approach to modeling RAS evolution in the context of SARS-CoV-2 infection. Inspired by a number of existing RAS models [35,36,37,38], we searched the literature for measured half-lives and concentrations of angiotensin peptides and their receptors in healthy normotensive and hypertensive individuals and then identified the unknown production and reaction rate parameters from the model. As an initial test, we compared our model predictions of how the administration of RAS-blocking drugs would affect Ang peptide concentrations and blood pressure with relevant experimental data; we found good quantitative agreement between our model and experimental data, without the need for further parameter fitting. We then modeled the effect of SARS-CoV-2 infection on RAS through the downregulation of ACE2, which we related to the SARS-CoV-2 viral load.

A focal point of our work was to investigate how a series of RAS-targeting drugs affected COVID-19 patients. We found that the administration of two antihypertensive drugs, ACE-I and DRI, tended to reduce the severity of COVID-19, while ARB drugs worsened it. Clinical data generally support the model’s predictions for the administration of ACE-I drugs, but they are either absent or partially contradict the model’s prediction for DRI and ARB administration. Additionally, we modeled a potential treatment that is currently under clinical trial in COVID-19 patients: administration of rhACE2 or Ang1-7 by drug infusion. Our model predicts improved clinical outcomes in these cases, in agreement with a series of experimental data on animal models.

It is important to note that, despite its simplicity, our model has excellent accuracy in reproducing clinical and experimental data on the perturbed RAS. Furthermore, the model’s predictions of changes in COVID-19 severity due to drug administration are blind predictions, without the fitting of any additional parameters.

Many challenges remain in our current understanding of RAS perturbation in COVID-19 patients. Importantly, more data regarding angiotensin peptide concentrations upon SARS-CoV-2 infection are urgently needed, since currently available data are often inconsistent or conflicting so that reliable comparisons between model predictions and experimental data cannot be made. Even in healthy individuals, angiotensin peptide levels can vary substantially due to their low circulating concentrations, the experimental techniques used to measure them, and inter-patient variability.

When developing our model, we chose not to consider two enzymes that are active in RAS through the cancellation of their reaction rates: CHY and NEP (see Equations (17) and (18)). The CHY enzyme is expressed in mast cells present in interstitial lung connective tissues, and it cleaves AngI to form AngII. the addition of this enzymatic reaction in the model would not really influence the predictions since it would essentially be a reparametrization of ACE activity and of ACE-I action. It might, nevertheless, be interesting to add the CHY enzymatic reaction, which yields ACE-independent synthesis of AngII and has been suggested (although debated) to be upregulated in the case of long-term ACE-I administration [78]; this would enable an explanation of why ACE-I fails to inhibit AngII formation after some time [78,79].

The NEP enzyme is expressed in a wide range of tissues, being particularly abundant in kidney, and it cleaves AngI to form Ang1-7. It influences the counterregulatory RAS axis through its connection to Ang1-7 levels, thus affecting COVID-19 severity. However, NEP’s role is far from clear, and the literature contains contradictory findings. Experimental data from rats with ARDS suggest that NEP is severely downregulated in both plasma and lung tissues [80]. Note that NEP also cleaves natriuretic peptides, which have both anti-inflammatory and anti-fibrotic effects [81]. Therefore, the combined administration of NEP-inhibiting and ARB drugs has been suggested to treat SARS-CoV-2 patients [82].

Our future work will include building more complexity into our model by explicitly considering the communication between local and systemic RASs [33,34] and by including the interaction between RAS and the immune system [83]. This model extension is necessary for an improved quantitative understanding of RAS dysregulation upon a variety of perturbations, including SARS-CoV-2 infection.

Our model and its predictions provide a valuable and robust framework for in silico testing of hypotheses regarding COVID-19’s pathogenic mechanisms and the effect of drugs that are aimed at restoring RAS functionality. Our work also opens a broader discussion on the role of the full RAS in COVID-19, a topic that has received little attention to date, perhaps due to the current focus on the ACE2 enzyme, which, although very important as it is directly targeted by the virus, constitutes only one part of a much more complex system.

## Figures and Tables

**Table 1:** Half-lives of the species involved in RAS and other parameters of the model. “Fitted” means fitted on experimental data.

Parameter	Unit	Values	Reference
$h_{agt}$	min	600	[35]
$h_{ang1--7}$	min	0.5	[35]
$h_{angI}$	min	0.5	[35]
$h_{angII}$	min	0.5	[35]
$h_{angIV}$	min	0.5	[35]
$h_{at1r}$	min	12	[35]
$h_{at2r}$	min	12	[35]
$h_{re}$	min	12	[35]
$h_{mas}$	min	12	-
$c_{re}$	1/min	20	[36,54]
$A_{0}$	mmHg	450	Fitted
$A_{1}$	mmHg	267	Fitted
$P_{0}$	mmHg	73.6	Fitted
$P_{1}$	mmHg mL/fmol	0.43	Fitted
*a*	-	0.53	Fitted
*b*	-	16.7	Fitted

**Table 2:** Equilibrium concentrations of the species involved in RAS and production and reaction rate parameters, for healthy normotensive and hypertensive humans. “Solved” means solved from the model.

Parameter	Unit	Normotensive	Hypertensive	Reference
[AGT]${}_{0}$	fmol/mL	6 $\times \;10^{5}$	6 $\times \;10^{5}$	[55]
[AngI]${}_{0}$	fmol/mL	70	110	[56,57]
[AngII]${}_{0}$	fmol/mL	28	156	[56,57]
[Ang1-7]${}_{0}$	fmol/mL	36	92	[56,57,58]
[AngIV]${}_{0}$	fmol/mL	1	1	[59]
[AT1R-AngII]${}_{0}$	fmol/mL	15	85	[37]
[AT2R-AngII]${}_{0}$	fmol/mL	5	27	[37]
[RE]${}_{0}$	fmol/mL	9.43	25.25	Solved
[MAS-Ang1-7]${}_{0}$	fmol/mL	6.43	15.92	Solved
$k_{agt}$	fmol/(mL min)	881.82	1198.22	Solved
$\beta_{0}$	fmol/(mL min)	0.54	2.21	Solved
$c_{ace}$	1/min	1.31	3.21	Solved
$c_{ace2}$	1/min	1.80	0.82	Solved
$c_{angIV}$	1/min	0.05	0.01	Solved
$c_{at1r}$	1/min	0.03	0.03	Solved
$c_{at2r}$	1/min	0.01	0.01	Solved

**Table 3:** Comparison between model predictions and experimental values of AngI and AngII levels normalized by their value before the administration of the drugs. Range is the interval of experimental values, and rmsd is the root mean square deviation between experimental and predicted values, computed over all time points; Np is the number of time points. ACE-I, angiotensin-converting enzyme inhibitor; ARB, angiotensin receptor blocker; DRI, direct renin inhibitor.

Drugs	Class	Dose	[AngI](t)/[AngI]${}_{0}$	[AngII](t)/[AngII]${}_{0}$	Np	Ref.
		(mg)	rmsd (Range)	rmsd (Range)		
Enalapril	ACE-I	20	1.31 [1.0–9.2]	0.09 [0.2–1.0]	5	[60]
Losartan	ARB	50	0.61 [1.0–2.1]	-	3	[61]
Valsartan	ARB	850	0.83 [1.0–2.2]	-	3	[61]
Irbesartan	ARB	150	0.97 [1.0–4.4]	-	3	[61]
Aliskiren	DRI	40	0.13 [0.4–1.1]	0.14 [0.5–1.0]	6	[62]
Aliskiren	DRI	80	0.15 [0.4–1.0]	0.16 [0.4–1.0]	6	[62]
Aliskiren	DRI	160	0.26 [0.2–1.0]	0.20 [0.3–1.0]	6	[62]
Aliskiren	DRI	640	0.29 [0.1–1.0]	0.29 [0.1–1.0]	6	[62]
**Mean**			**0.57**	**0.18**		

**Table 4:** Prediction of biochemical and clinical features of SARS-CoV-2 patients.

	Uninfected	Mild	Moderate	Severe
$C_{t}$	40.0	31.5	27.6	23.8
Normotensive				
[AngII] (fmol/mL)	28	32	36	38
[Ang1-7] (fmol/mL)	36	21	5	1
PaO2/FiO2 (mmHg)	450	300	145	98
DBP (mmHg)	80	81	82	82
Hypertensive				
[AngII] (fmol/mL)	156	186	221	231
[Ang1-7] (fmol/mL)	92	55	15	2
PaO2/FiO2 (mmHg)	450	292	115	60
DBP (mmHg)	110	117	125	128

**Table 5:** Predicted effects on AngII and Ang1-7 levels, PaO2/FiO2, and DBP upon drug administration to normotensive and hypertensive COVID-19 patients. the drug administrations are modeled by $\gamma_{ACE-I},\gamma_{\mathrm{ARB}},\gamma_{\mathrm{DRI}},\gamma_{rhACE2}=0.5$, and $\eta_{Ang17}=25$ fmol/(mL min) and moderate SARS-CoV-2 infection by $\gamma_{CoV}=27.6$.

Drugs	No Drugs	ACE-I	ARB	DRI	rhACE2	Ang1–7
Normotensive—Moderate Infection						
[AngII]/[AngII]${}_{0}$	1.29	1.10	1.98	0.99	1.10	1.29
[Ang1-7]/[Ang1-7]${}_{0}$	0.15	0.13	0.23	0.11	0.68	0.64
PaO2/FiO2 (mmHg)	145	188	0	216	337	278
DBP (mmHg)	82	81	80	80	81	82
Hypertensive—Moderate Infection						
[AngII]/[AngII]${}_{0}$	1.42	1.12	1.55	0.77	1.14	1.42
[Ang1-7]/[Ang1-7]${}_{0}$	0.16	0.13	0.18	0.09	0.70	0.36
PaO2/FiO2 (mmHg)	115	185	83	268	332	167
DBP (mmHg)	125	114	101	102	115	125

## Data Availability

The code used to generate all the results of this paper is freely available on the GitHub repository (https://github.com/3BioCompBio/RASinCOVID).
